# Supplementary material for: Are carbon emissions trading and green financial instruments synergistic? -Comprehensive quantitative research based on content analysis
Source: PLoS One. 2024 Mar 7;19(3):e0298601. doi: 10.1371/journal.pone.0298601 (PMC10919601; doi:10.1371/journal.pone.0298601)
Supplement: S1 Data — (ZIP) [file pone.0298601.s001.zip › Data/地级市Z_F工作报告.zip!/┴╔─■/╜⌡╓▌╩╨/└┤╫╘╣1⁄2╓┌║┼í╛┬φ┐╦╩2╛▌═°í┐_2017.docx]

各位代表：

　　现在，我代表市政府向大会报告工作，请予审议，并请市政协各位委员提出意见。

　　一、2016年工作回顾

　　过去一年，锦州发展面临多重困难和严峻挑战。在省委、省政府和市委坚强领导下，全市人民以坚定的信心和顽强的斗志，迎难而上，奋力拼搏，统筹推进稳增长、促改革、调结构、惠民生、防风险各项工作，保持了经济平稳发展、社会和谐稳定。

　　初步预计，全市实现地区生产总值1035.1亿元，固定资产投资379.8亿元，一般公共预算收入84.8亿元，社会消费品零售总额610.7亿元，出口总额10.35亿美元，城镇和农村常住居民人均可支配收入分别达到28484元和13539元。

　　（一）经济稳增长措施扎实有效

　　全面落实新一轮东北振兴政策。贯彻中央7号文件、国务院28号文件精神，细化分解127项具体任务。松山新区入选首批国家级产城融合示范区，黑山获批国家农村产业融合发展试点示范县，北镇沟帮子被列为国家新型城镇化综合试点地区。全市争取上级各类资金260亿元。推出PPP项目31个，市文化艺术中心、锦州师专迁建等7个项目进展顺利。总规模16亿元的锦州医疗健康产业基金正式签约。积极开展区域合作，与盘锦市签订交流合作框架协议。

　　招商引资项目建设年成果显著。全市上下积极走出去、请进来，引进总投资3000万元以上项目241个，实际到位省外资金172.5亿元。新组建两个驻外招商局。全市在建亿元以上项目200个，完成投资246.2亿元。华润锦州电厂热电机组等一批投资10亿元以上项目开工建设，沃特玛联盟新能源汽车产业园、奥鸿药业生物制药产业园等重大项目顺利推进。

　　积极应对经济下行压力。加强对100户重点企业运行监测分析，对60户企业实行市级领导定点帮扶。举办地工产品产销衔接会10次。采取多种措施降低企业运营成本，“营改增”为企业减负4.8亿元，组织银企对接实现信贷签约15.6亿元。表彰功勋企业50户，激励企业为稳增长做贡献。

　　大力培育信息、旅游休闲、教育文体和健康养老家政等消费热点。实现展会交易额 41.6亿元，电子商务交易额增长20%。现代物流、连锁经营等新业态竞相发展，农业生产流通服务体系逐步健全。古塔太阳广场、凌河茂业大厦、太和盛世汽车交易中心等重点商业项目加快建设。旅游总收入增长14.1%。我市及北镇获批国家全域旅游示范区创建单位，北镇还获评省全域旅游示范县。成功举办古玩文化节、温泉冰雪节等大型节展活动。

　　对外贸易呈现新增长点。华泰锦州公司成功申领500台汽车出口许可证。锦州港获批公用型液体危险品保税仓库资质，规划年进出口额4亿美元。2家央企国贸子公司在锦注册。培育海外仓10个，设立境外投资企业5家，新列入省重点培育自主出口品牌产品2个。

　　（二）两个先导区引领作用不断增强

　　“以港兴市”战略深入实施。抢抓“一带一路”机遇，与锡盟和蒙古国交流合作持续深化。中蒙铁路新通道出海口建设纳入省“十三五”规划，锦赤铁路全线铺通进行重载试运。锦州港经二连浩特至蒙古国过境油品业务获海关总署批复，蒙古国海事局锦州代表处成立。推进“三互”大通关建设，通关便利化水平不断提高。口岸全面开放、保税物流中心（B型）报批工作进展顺利。锦州湾机场增开锦州-天津-海口、昆明-锦州-哈尔滨航线。

　　国家级开发区开放牵动作用凸显。大学城成为滨海新区人文新坐标，入驻院校5所，在校师生2.2万人。北医三院锦州分院医养结合中心等项目签约。临港产业快速发展，华泰新能源汽车生产基地、威涛电商物流园等项目开工建设，万得汽车电器产业园、中铁北方生产基地等项目投产，军民融合产业园3家企业入驻。盘活闲置厂房36.7万平方米。

　　国家高新区展现新形象。大学科技园起步区14幢楼主体竣工，北航大学科技园锦州分园、华为锦州云数据中心等10个亿元以上项目签约。国家科技成果转化服务(锦州)示范基地信息平台进入调试阶段。万得总部大厦等项目开工建设，环宇科技集成电路产业园等项目建成投产，万达广场、红星美凯龙家居广场等服务业项目建设进展顺利。

　　（三）产业结构调整步伐加快

　　全力推进工业“6921”、传统产业转型升级、《中国制造2025》提升和质量品牌建设四项工程实施。天晟重工大型电石炉进入国家首台（套）重大技术装备推广应用指导目录。锦州钛业等3户企业入选国家两化融合贯标试点。汉拿电机等4户企业晋升省智能制造试点示范企业。获评省级名牌产品8个、著名商标8个、“专精特新”产品（技术）14个。新认定国家高新技术企业14家。新组建省级工程技术研究中心2家、重点实验室2家、产业专业技术创新平台4个、产业技术创新联盟2个。创建国家级众创空间2个、省级3个。评选第四批市学术和技术带头人173名。智慧城市建设持续推进。

　　现代服务业发展势头良好。滨海国家电子商务示范基地获评“全国十佳电子商务园区”。锦州电子商务、创新创业两大公共服务平台在全省率先上线运行。黑山农村淘宝、北镇方大、凌海展望科技、义县村邮乐购等农村电子商务平台快速兴起。辽西区域金融中心加快建设，华夏银行、朝阳银行在锦设立分支机构，锦州农商银行挂牌营业，春光包装等3户企业在“新三板”挂牌。出台助企惠民政策，举办春夏秋三季房交会，商品房销售165万平方米，库存去化周期仅15个月，居全省第一。

　　现代农业加快发展。粮食总产量达到49.9亿斤。建设高标准农田16.9万亩、高效节水灌溉9.88万亩，新增“三品”生产基地认证面积60万亩。培育省级现代农业设施小区16个。耕种收综合机械化水平达到84%。完成农村土地承包经营权确权登记颁证面积430万亩。建成益农信息社792家。新增农民合作社399个、备案家庭农场54个。培育标准化养殖场12个，规模化养殖率65%。黑山成为辽育白牛繁育基地。创建农业部水产健康养殖示范场3家。主要农产品监测合格率95%。

　　（四）全面深化改革实现新突破

　　持续推进“放管服”改革。取消、下放和调整行政审批事项103项，削减率22.9%。成立市行政审批局，在全省率先启用审批专用章。优化审批流程，招商和政府投资类项目审批分别提速43%和27%。网上审批服务平台投入运行。建立权责清单动态调整机制，减少行政职权225项。全面实施“五证合一、一照一码”，新发展市场主体2.6万户。

　　国资国企改革不断深化。组建了粮食、顺达资产、交通、旅游等8个企业集团。完成市级党政机关与所属企业脱钩改制。驻锦央企“三供一业”分离工作进展顺利。对8户全民所有制企业实施公司化改革。破产、注销“僵尸”企业10户。

　　政府债务和养老金管理进一步规范。出台实施意见，推进债券发行和置换，债务结构持续优化，风险指标得到有效控制。严格收支管理，多渠道筹措资金，保证了养老金按时足额发放。

　　深化产业园区体制机制创新，建立“管委会+公司”管理体制。稳步推进事业单位改革，撤销、整合市直事业单位14家。完成机关公务用车改革。机关事业单位养老保险、供销合作社、国有林场改革取得新进展。

　　（五）城乡环境面貌明显改观

　　城市规划建设管理进一步加强。编制完成渤海大道城市带等专项控制性规划，严格规划审批程序和刚性执行。组建了城市公园管理局。南山公园一期竣工开放，成为锦州又一张靓丽的名片。城市楼体亮化工程让锦城夜色更美。锦凌水库分部工程通过验收，净水厂主体工程完工。全年改造城市街路47条，施划城区静态停车位1.6万个。城市精细化管理水平不断提高。

　　农村环境建设整治成果稳固提升。宜居乡村建设投入资金3.9亿元。新建文化广场155个，村内道路硬化665公里，改造农厕2.5万户。新建改建农村公路396公里，建设农村客运候车亭62个。新建宜居示范村138个。

　　生态环境进一步改善。拆除燃煤小锅炉589台，淘汰黄标车及老旧车辆14526台，秸秆禁烧责任制全面落实。人工造林15.1万亩，封山育林13.6万亩，治理水土流失31万亩。新增新能源公交车210台。中科垃圾焚烧厂、城南热源厂具备运营条件。PM10、PM2.5浓度分别改善12.4%和7.1%，域内主要河流水质稳定达标。

　　海岸带专项整治成效显著。海洋管理进一步规范，岸线环境质量明显提升，海洋生态建设全面启动，开发开放氛围日益浓厚，全新生态文明海岸带正在逐步形成。

　　（六）社会民生事业持续改善

　　全力脱贫攻坚。投入财政扶贫资金9200万元，严格落实精准扶贫，实现农村2.7万人脱贫、54个贫困村销号。省产业扶贫现场会在义县召开。

　　就业形势总体稳定。城镇新增就业1.78万人，登记失业率控制在3.92%以内。发放创业担保贷款4.5亿元。辽西区域性人力资源市场投入使用。

　　社会保障更加有力。继续上调企业退休人员基本养老金和城乡低保标准。新农合补助标准达到420元。棚改开工率在全省率先实现100%，基本建成4363套，为年计划的433%。整治老旧小区330万平方米。市养老综合服务中心投入使用，填补我市大型公办养老机构空白。完成1118户贫困残疾人家庭个性化无障碍改造。解决农村8.2万人饮水安全问题。

　　基础教育建设年持续推进。凌海、黑山、太和、滨海新区“义教均衡”通过省验收，其中凌海通过国家认定。锦州中学新校区建设顺利推进。普惠性幼儿园学位覆盖率达65%，改善了80所农村义务教育薄弱学校办学条件，中等职业学校整合成效不断显现。

　　文化建设年成果丰硕。精品佳作好戏连台，新编复排剧目9部，举办大型专题晚会10场。开展“凌河之夏”演出季等一系列文化惠民活动，组织大型公益演出150余场、公益讲座近百场，群文作品入围全国第十一届艺术节“群星奖”。万人文化广场投入使用。完成辽沈战役纪念馆基本陈列升级改造。南庄里文创城等文化产业示范园建设加快推进。

　　健康锦州迈出新步伐。城市公立医院综合改革试点全面启动。市中心医院门诊楼加快建设，医学影像中心、生殖中心投入运营。发放居民健康卡166万张，建成公共场所哺乳室100家。优化整合县域乡镇卫生计生资源，城乡分级诊疗体系初步建立。“两孩”政策全面实施。在全省首批实现职工医保省内异地结算。

　　体育事业蓬勃发展。成功举办和承办市第二届全民健身体育运动大会、省第七届老年人运动会等重要体育赛事，万人登山等群众性体育运动影响广泛。

　　食品药品安全专项整治成效明显。突出问题得到有效解决，长效监管机制逐步建立，保障了全市人民“舌尖上的安全”。义县食品安全应急管理示范县通过省考核验收。

　　平安锦州建设取得新进展。立体化社会治安防控体系更加完善，维护稳定、打击整治、服务发展能力显著提升，群众安全感明显增强。修订完成《锦州市突发事件总体应急预案》。医巫闾山和北普陀山森林防火预警系统投入使用。市群众诉求服务动态监管平台建成运行，率先在全省实现诉求渠道全面整合。91件涉众型历史遗留信访积案全部办结，上级交办积案办结率100%。扎实开展煤矿、油气管道、道路交通等隐患专项整治，安全生产形势总体稳定。成功防御7····20、7····25强降雨，气象服务和防灾减灾能力不断增强。民族团结进步创建深入开展。锦州获得全国双拥模范城“四连冠”。

　　(七)政府自身建设全面加强

　　强化政府系统党的建设。市政府党组以上率下，扎实开展“两学一做”学习教育，以党章为根本遵循，以辽宁拉票贿选案为镜鉴，牢固树立“四个意识”，认真做好中央巡视“回头看”反馈意见整改落实，坚定自觉地在思想上政治上行动上同以习近平同志为核心的党中央保持高度一致，确保了党的路线方针政策在锦州落地见效。

　　严格遵守政治纪律和政治规矩。坚持重要事项提请市委常委会议审议，坚决落实市委部署要求。主动接受人大法律监督、工作监督和政协民主监督，认真听取各民主党派、工商联、无党派人士和各人民团体意见。142件人大代表建议、206件政协提案全部办复。

　　法治政府建设迈出新步伐。严格执行政府决策议事规则，全面规范行政执法。推进重点领域政务公开，坚持重大事项社会听证、公示。依法完成第十二届村委会换届选举。创新工作体制，实现“督考合一”，绩效考评“风向标”“指挥棒”作用更加凸显。人民调解、公证服务不断加强，锦州获评“全省法治宣传教育先进城市”。

　　落实全面从严治党主体责任，自觉履行“一岗双责”。聚焦五大方面50个问题，全力开展软环境集中整治。成立软环境建设工作领导小组办公室, 与机关作风监督局合署办公。强化行政监察和审计监督，严肃查处一批违法违纪案件。认真贯彻中央八项规定和省、市细则，公务用车、办公用房、政务接待等更加规范，政府系统风清气正、干事创业氛围日益浓厚。

　　国家安全、防震减灾、海防、人防和民兵预备役等工作不断加强，外事、侨务、台务、档案、慈善、住房公积金等工作取得新成果。

　　各位代表，过去一年的成绩来之不易，面对严峻复杂的外部环境和经济持续下行压力，我们经受住了一系列挑战和考验。我们深切感受到，成绩的取得，是省委省政府和市委总揽全局、正确领导的结果，是市人大市政协认真履职、有效监督的结果，是全市人民齐心协力、共同奋斗的结果。在此，我代表市政府，向全市人民表示崇高的敬意！向人大代表、政协委员，向各民主党派、工商联、无党派人士和各人民团体，向离退休老同志，向驻锦中省直单位和解放军指战员、武警官兵，向所有关心支持锦州振兴发展的海内外朋友，表示诚挚的感谢！

　　在回顾去年成绩的同时，我们也清醒看到，锦州发展中还存在不少困难和问题：一是受经济下行压力持续加大，以及我市产业结构不合理、产品结构不适应市场变化等诸多因素影响，个别经济指标未能完成预期。二是招商引资力度还不够大，落地建设的重大项目较少，盘活存量、提质增效办法不多创新不够。三是财政收支矛盾加剧，社会保障压力较大，一些领域存在风险和隐患。四是在深入落实国家和省振兴政策上还有一定差距，个别干部缺乏应有的工作激情，不敢担当、不愿作为。五是一些行业和领域侵害群众利益的不正之风和腐败问题不容忽视，软环境建设需要持续发力，党风廉政建设还需不断加强。我们将进一步增强忧患意识，下更大力气解决这些问题，始终以民之所望为施政所向，尽心竭力做好政府工作，决不辜负全市人民的重托。

　　二、2017年主要工作安排

　　各位代表，今年是党的十九大召开之年，是全面实施“十三五”规划的重要一年，也是推进供给侧结构性改革的深化之年。做好新一年的工作，我们充满必胜信心。中央实施新一轮东北振兴战略，为我们带来了重大的政策机遇；中央巡视组反馈意见的全面整改落实和全省政治生态的修复与重塑，为我们提供了坚强的政治保障；市第十二次党代会的胜利召开，为我们描绘了宏伟的发展蓝图。特别是经过多年建设发展，锦州加快振兴已经具备坚实的基础，勤劳智慧的锦州人民求发展、盼振兴的正能量正在加速凝聚。

　　优势在握，机遇在前。我们必须牢牢抓住发展第一要务不放松，努力实现项目建设的重大突破；必须大力推进结构性改革，努力实现发展动力和市场活力的竞相迸发；必须把民生为本的理念念之再三、铭之肺腑，努力让全市人民的生活更加富足幸福。信心就是力量。只要我们坚定发展信心，保持战略定力，求真务实、埋头苦干，锦州振兴发展的步伐一定会更加铿锵有力。

　　今年政府工作的总体要求是：以党的十八大和十八届三中、四中、五中、六中全会以及中央经济工作会议精神为指导，深入贯彻习近平总书记系列重要讲话精神，统筹推进“五位一体”总体布局，协调推进“四个全面”战略布局，抢抓新一轮东北振兴机遇，认真落实省、市党代会和经济工作会议精神，适应把握引领新常态，持之以恒践行新发展理念和“四个着力”要求，坚持稳中求进工作总基调，坚持以提高发展质量与效益为中心，以推进供给侧结构性改革为主线，以保障和改善民生为根本，全面营造良好发展环境，深入实施“五大战略”，加快打造实力、法治、文明、生态、幸福锦州，奋力开创振兴发展新局面。

　　今年发展的主要预期目标是：地区生产总值增长6.5%，固定资产投资增长12%，一般公共预算收入增长5%，规模以上工业增加值增长7%，社会消费品零售总额增长9%，出口总额增长5%。居民消费价格涨幅控制在3%。城镇新增就业1.6万人，登记失业率控制在5%以内。城镇和农村常住居民人均可支配收入均增长7%。

　　确定以上目标增幅，既考虑与“十三五”规划和市第十二次党代会总体目标相衔接，又考虑与省委省政府加快推进全面振兴的部署要求相一致。在具体工作中，我们将向更高的目标努力，力争实现更好更快发展，确保主要经济指标总量保持在全省应有位次、增幅不低于全省平均水平。

　　要实现今年发展目标，面临很多挑战和压力，但我们决不能退缩、决不能懈怠，必须顶住压力、迎难而上，坚决贯彻省委省政府的部署要求，团结一心、苦干实干，确保全年目标任务圆满完成。

　　今年要重点做好九个方面工作。

　　（一）全面落实振兴发展政策举措，再掀项目建设新高潮

　　全力确保中央新一轮东北振兴政策落地生效。继续抓好中央7号文件、国务院28号和62号文件、《东北振兴“十三五”规划》政策举措的贯彻实施，结合落实省促进振兴发展系列部署，逐项明确责任，全程跟踪问效，加快推进经济企稳向好。

　　深入开展招商引资项目建设年活动。深化招商体制改革，探索公司化招商新模式，建立各级领导带队招商机制。瞄准京津冀、长三角等重点区域，围绕产业集群、生产基地和新兴产业，广泛开展产业链招商、以商招商和专业招商。咬住全市引进内资360亿元、外资3.5亿美元的目标不动摇。狠抓责任落实，制定任务分解方案，明确责任单位、责任人，落实时间表和路线图；狠抓项目调度，继续推行重大项目市领导包扶制度，坚持问题导向，对重大项目按月调度，按时间节点推进；狠抓考核问责，既考核引进项目数量、质量与投资额度，更考核签约项目履约率、开工率和投产率。全年计划安排亿元以上项目191个，完成投资246亿元。重点推进邈矣集团锦州港石化、沃特玛联盟新能源汽车锦州产业园、国测石墨科技产业园、古塔精细化工产业园等一批重大项目，形成新的投资规模和增量。

　　提升外贸进出口水平。深化与重点国贸企业合作，着力推进东电三公司与长征集团合作建设孟加拉广播电视台、万得集团并购国外知名汽车零部件企业等项目实施。开展“百企百展”工程，拓展出口市场。加强出口基地建设，培育10-15家年出口额超千万美元骨干企业。

　　（二）加快两个先导区建设，打造振兴发展新增长极

　　推动海港空港建设发展。加快推进中蒙俄铁路新通道出海口建设。实现锦州港口岸全面开放，力争设立保税物流中心（B型）。深化“三互”大通关改革。开展《锦州港总体规划》修编，完成港口建设投资10亿元。锦州湾机场新增航线2条，推进空港物流园区建设。

　　提升国家级开发区综合竞争力。加快壮大滨海新区大学城规模，重点推进锦州师专、锦州医科大学、锦州医疗学院迁建。加速产业集聚发展，华泰新能源汽车、军民融合产业园入驻企业等项目建成投产，加快推进国电投氧化铝、神雾能源等项目前期工作。完善城市功能，力促北医三院医养结合中心、北大附属实验学校、滨海客运枢纽站等项目开工建设。

　　提升国家高新区建设水平。确保大学科技园起步区投入使用，北航大学科技园锦州分园等10个项目开工建设，万得总部大厦及汽车研究院等项目竣工。加快松山金融集聚区建设，实现科技金融服务中心试运行。确保万达广场等服务业项目投入运营。用足用好国家产城融合示范区政策，建立健全工作推进机制，加快形成以产兴城、以城带产、产城融合发展新格局。

　　（三）推动工业结构调整，提升经济运行质量和核心竞争力

　　全面贯彻省工业供给侧结构性改革实施意见，细化落实我市装备制造、石化、冶金建材、消费品、电子信息等五大产业发展政策，制定实施培育和发展新兴产业三年行动计划，以产业链为中心，加快补齐产业发展短板。

　　深入实施工业稳增长等“工业强市”五项工程。强化对100户重点企业运行监测分析，坚持市级领导帮扶重点企业，举办地工产品产销衔接会，有效搭建银企对接平台，积极拓展“助保贷”业务，帮助企业解决市场、资金等实际问题，培育规下、规上企业做大做强，全力促进工业经济企稳向好。推动20户龙头企业与100户成长型企业协同发展，增强产业丰厚度。全年实施工业项目450个，着力推进德瑞克汽车配件等一批重点项目开复工，奥鸿药业生物制药产业园等竣工项目投产达效，重点培育瑞光电子集成电路等50个新兴产业项目。推进智能制造，培育“数字车间”10个。新增省市级企业技术中心5个、“专精特新”产品（技术）8个，创省市级名牌产品10项。

　　打造辽西区域创新研发中心。搭建各类创新创业平台，组建一批工程技术研究中心、重点实验室、产业技术创新战略联盟和众创空间。实施企业创新主体培育“双百”工程，实现高新技术企业和高成长性科技型中小企业数量大幅增长。推进与中科院沈阳分院合作，支持辽宁工业大学创建国家级大学科技园。充分发挥驻锦高校资源优势，加速产学研协同创新，促进科研成果本地转化。加快智慧城市建设进程。

　　（四）深入推进农业供给侧结构性改革，提高县域经济综合实力

　　加快发展现代农业。严守耕地红线，实施藏粮于地、藏粮于技战略，粮食产量稳定在50亿斤以上。调减玉米种植面积15.1万亩。建设标准化设施农业小区10个，打造一批特色鲜明、集中连片的农产品生产基地。力争获批1个国家级农业标准化示范区。建设高标准农田17.7万亩，节水灌溉9万亩。继续开展土地经营权确权登记颁证工作，有序推进土地流转。培育新型农业经营主体和服务主体，大力发展观光休闲农业等新业态。实施“互联网+惠农工程”，推进信息进村入户，益农信息社覆盖率80%。力保不发生农产品质量安全重大事件和区域性重大动植物疫情。加快农村电网升级改造。耕种收综合机械化水平达到86%。

　　扎实开展产业园区建设年活动。巩固产业园区体制机制创新成果，出台管理考核办法，确保“管委会+公司”管理体制稳定运行。力争实现省级以上县域产业园区审批不出园区。启动重点园区总体规划修编，调整完善园区产业发展规划，依托资源禀赋、区位优势，因地制宜培育壮大一批特色产业集群。加强园区空间建设，盘活闲置厂房，提高土地利用效益。力促大有经济区、汤河子工业园区晋升省级开发区。

　　推进新型城镇化。促进城乡规划、建设和基本公共服务一体化。加快培育发展一批特色小镇。继续巩固提升农村环境整治成果，健全常态化保洁机制，完善农村基础设施，实现宜居乡村建设达标村全覆盖。

　　（五）大力发展现代服务业，激发城区经济发展潜力

　　落实服务业供给侧改革政策措施，推动生产性服务业专业化高端化、生活性服务业优质化便利化，满足社会生产生活新需求。

　　促进商贸流通业转型升级。加大培育力度，新增10家限额以上批零住餐企业。开展百店大促销活动，推进锦州烧烤特色产业集聚发展。加强与会展企业合作，推出更多品牌展会。依托区位优势，支持综合物流园区建设，加快发展冷链物流、多式联运、共同配送等新模式。培育中国北方杂粮物流交易中心等大宗商品现货交易市场。利用各类电商服务平台，大力发展跨境电子商务。推进省电子商务云数据中心等项目建设。与阿里巴巴等知名电商合作建立“特色馆”，把锦州更多特色农产品推向全国。

　　扎实推进国家全域旅游示范区创建工作。加大北普陀山、锦凌水库及小凌河城区段至世博园沿岸旅游规划开发力度。做好医巫闾山、辽西九华山5A级景区争创工作，改造完善辽沈战役纪念馆等景区环境。办好梨花节、冰雪节等特色节展活动。强化区域合作、品牌创建和宣传推介，推进旅游服务集散中心建设。旅游总收入增长12%。

　　加快辽西区域金融中心建设。全力推进锦州银行（A股）等企业上市工作，力争3户企业在“新三板”挂牌、2家域外金融机构在锦落户。逐步推进四县（市）农信社改制。

　　各城区要依托产业优势，进一步明确功能定位，突破瓶颈制约，通过差异化、特色化发展，激发现代服务业发展潜能，构筑城区经济发展新格局。

　　（六）持续深化各项改革，不断释放经济发展新活力

　　全面落实《推进供给侧结构性改革实施方案》，制定各领域专项方案，深入推进“三去一降一补”。去产能方面，支持企业通过技术改造、兼并重组等方式化解过剩产能。去库存方面，继续调整去化周期，商品房销售面积增长10%。去杠杆方面，发挥高新技术产业投资基金、医疗健康产业基金等引导带动作用，吸引社会资本投向优势企业。降成本方面，落实税费减免政策，降低企业用能、物流及各类交易成本，引导企业提升管理水平。补短板方面，从严重制约经济社会发展的重要领域和关键环节入手，采取切实有效的措施和办法，着力补齐脱贫攻坚、科技创新、优化结构、改善生态、强化公共服务等方面的发展短板和制度短板。

　　深化“放管服”改革。确保市行政审批局规范有序高效运行，切实做到“一个窗口受理、一站式办理、一条龙服务”。完善权责清单等制度，建立长效机制。适时开展企业投资项目承诺制试点。推进网上审批服务平台互联互通，加快打造审批服务升级版。6月底前完成各县（市）区行政审批局组建。建设政府协同办公系统。推进“多证合一”等登记制度改革，实现“双随机、一公开”监管全覆盖。

　　深化公共资源交易管理体制改革。整合公共资源交易平台，出台管理办法及交易目录，实现法制化、规范化、透明化运作，提高公共资源配置质量和效益。

　　加大国资国企改革力度。对新组建企业集团进行资源整合优化配置，实现国有资产保值增值。对华信集团和城投公司实施去平台化改革。完成驻锦央企“三供一业”分离移交。稳步推进厂办大集体改革。积极探索事业单位分行业改革，推进经营性事业单位转企改制。完成事业单位公务用车、国有林场改革。有序推进供销社综合改革。

　　开展优化营商环境建设年活动。从完善体制机制入手，实施优化投资营商环境专项行动，按照省部署要求，制定具体工作方案，切实解决影响经济社会发展各类软环境问题。落实扶持民营经济发展政策措施，加快推动民营经济发展。广泛运用PPP等融资模式，吸引更多社会资本参与锦州发展建设。

　　强化政府债务管控。继续实行政府债务规模控制限额管理，做好债券发行置换等工作，保证政府债务风险可控。加强养老金收支管理，确保按时足额发放。

　　（七）统筹推进城乡协调发展，加快建设生态锦州

　　坚持规划先行。启动城市总体规划修编，开展总体城市设计和特色风貌研究。开工建设城市规划展示馆。

　　实施城市畅通工程。启动锦州绕城公路新建工程。开工建设广州街小凌河大桥、中环西路续建及滨河路延伸工程。改造徐州街，建设地下农贸市场。实施锦州南站广场改造和汽车客运南站新建工程。新建大修街路30条，有序打通部分断头路。加强静态车辆管理。

　　实施城乡小街小巷建设工程。努力实现全市小街小巷路面平、排水畅、设施全、路灯亮，规范整洁，优美宜居。

　　实施景区建设改造工程。强化城市公园建设管理，启动建设南山公园二期，为市民提供更好休闲运动空间。

　　实施城市美化亮化工程。完成四个高速出入口景观美化亮化。抓好“两路一街”“两河两岸”亮化工程，让锦城的夜空更加绚丽多彩。

　　着力提升城市精细化管理水平。开展城区街路“五乱”系列专项整治，细化落实网格化监督管理职责。

　　实施环境保护和建设三年行动计划。全面落实“水气土”十条。加大陆源污染治理和农业面源污染防治力度，提升水环境质量。拆除燃煤小锅炉167台，淘汰黄标车及老旧车辆5623台，全面落实秸秆禁烧责任制。加快大气污染防治地方立法进度。落实土壤污染防治方案，建设更多生态乡村。人工造林10.6万亩，封山育林7万亩，治理水土流失30万亩。实施干旱地区森林防火综合治理项目建设。完成混合垃圾填埋场工程，推进南山垃圾填埋场封场和垃圾二级转运站建设。

　　实施海岸带生态建设三年行动计划。统筹推进“十大行动”，实施锦州蓝色海湾整治项目，创建大笔架山、大凌河口两个国家级海洋公园，建设国家级海洋牧场，加快打造环境优美、生态宜居、产业兴旺的黄金海岸。

　　（八）推动各项社会事业进步，尽心尽力办好民生实事

　　全市人民对美好生活的向往，就是我们的奋斗目标。虽然财政收支矛盾加剧，但该给全市人民办的实事一件也不能少。

　　坚定不移推进脱贫攻坚。坚持以产业扶贫为主导，加大行业部门扶贫力度，建立精准脱贫长效机制，坚决完成2.5万人脱贫、56个贫困村销号年度任务。全力支持义县脱贫攻坚。

　　保持就业形势总体稳定。实施更加积极的就业政策，重点抓好高校毕业生、就业困难群众等重点人群就业。坚持创业带动就业，发挥人力资源市场作用，多渠道稳定和扩大就业，解决就业矛盾。

　　不断完善社会保障体系。进一步提高城乡低保和特殊困难群众救助标准。城乡居民养老保险和新农合参保覆盖率均超过90%。棚户区改造4464套，基本建成600套。加强市养老综合服务中心管理，开展关爱老年人健康工程。争创国家级综合减灾示范区2个。对930户贫困残疾人家庭进行个性化无障碍改造。推进社区减负和城市社区“十分钟便民服务圈”建设。

　　完成基础教育建设年任务。推进学前教育公益普惠发展，普惠性幼儿园学位覆盖率达到68%。义县和松山新区接受省级“义教均衡”评估验收。完成锦州中学搬迁。推进职业教育产教融合、校企合作。“三通两平台”全面建成投入使用。

　　扎实开展医改攻坚年活动。推进医疗、医药、医保联动改革，全市公立医院取消药品加成实行零差率销售。完善基层首诊、双向转诊、分级诊疗体系。完成市级区域卫生信息平台建设。加强重大疾病预防控制，推进卫生计生服务均等化。加快医养结合大健康产业发展。筹备创建国家卫生城。

　　持续推进文化建设年活动。加强精品剧目创作，打造群众文化节、京剧票友节等特色品牌，举办辽西五市首届民俗文化节。开展文化惠民演出100场。推进闾山辽陵考古发掘，做好奉国寺申遗工作。完成市文化艺术中心主体工程，开工建设市档案馆新馆。推进智慧广电发展，建设无线城市。培育壮大一批特色文化产业示范基地。

　　加快体育事业发展。承办好全国青少年田径锦标赛等国家级赛事，推进城区15分钟健身圈建设和体育场馆对外开放，推动体育产业发展。

　　实施食品药品安全整治三年行动计划。坚决贯彻习近平总书记“四个最严”要求，强化部门监管，落实企业主体责任，夯实基层基础，加大宣传和打击力度，构建科学完善的食品药品安全治理体系，严守每一道防线，让全市人民吃得安全、吃得放心。

　　推进平安锦州建设。加强立体化、信息化社会治安防控体系建设，实施派出所基础建设三年规划，进一步推进规范执法，依法惩治各类违法犯罪行为，实现打击整治常态化。实施“七五”普法规划，扎实开展法治宣传教育，提升法律援助和人民调解工作水平。严格落实安全生产“党政同责、一岗双责、齐抓共管”责任制度，强力开展隐患排查治理，坚决遏制重特大安全事故发生。深化信访工作制度改革，依法分类处理各类信访诉求，再化解处理一批信访积案。强化应急管理、网络安全和防灾减灾体系建设。加强和改进新形势下民族宗教工作，促进民族团结、宗教和谐。扎实做好双拥共建，巩固军政军民团结良好局面。

　　（九）提升行政效能和服务水平，建设人民满意政府

　　全面加强政府系统党的建设。深入贯彻党的十八届六中全会精神，牢固树立“四个意识”，严肃党内政治生活，强化党内监督制度，切实承担起全面从严治党主体责任，有效发挥政府系统各级党组织的政治核心作用。全面落实《锦州市落实省第十二次党代会精神任务分解方案》，切实用党代会精神统一思想、凝聚力量，汇聚推进锦州振兴发展的强大合力。

　　建设法治政府。严格遵守宪法和法律，自觉用法治思维推动工作。依法接受人大法律监督、工作监督和政协民主监督，坚持重大事项向人大报告和政协通报制度，坚决执行人大及其常委会各项决议、决定，积极支持人民政协履行职能，认真办理人大议案、代表建议和政协提案。改进政府立法，加强执法监督，推进政务公开，让权力在阳光下运行。

　　建设廉洁政府。坚持把纪律挺在前面，严格执行中央八项规定和省、市细则，持之以恒加强作风建设，严厉整治各种顶风违纪行为，坚决纠正各种侵害群众利益的不正之风。严肃行政监察、审计监督和执纪问责，持续营造良好政治生态。

　　建设高效政府。政府系统工作人员要把振兴发展的使命扛在肩上，把万家忧乐放在心头，恪尽职守、夙夜在公，主动作为、善谋勇为，不断提高履职能力。要健全并严格工作责任制，强化“督考合一”，严厉整治弄虚作假、不担当、乱作为等庸政懒政怠政行为，确保各项政策和任务不折不扣落到实处。

　　各位代表！奋斗成就梦想，实干赢得未来。让我们紧密团结在以习近平同志为核心的党中央周围，坚定不移地贯彻中央和省各项决策部署，在市委的坚强领导下，振奋精神，顽强拼搏，为加快辽西区域中心城市建设而努力奋斗，以优异成绩迎接党的十九大胜利召开！
